# Contractions of the Uterus Throughout Pregnancy

**Published:** 1887-10

**Authors:** J. Braxton Hicks

**Affiliations:** London, England


THE BUFFALO Medical AND Surgical Journal

VOL. XXVIII. OCTOBER, 1887. No. 3

### Original Communications.

### *NINTH INTERNATIONAL MEDICAL CONGRESS.

HELD AT WASHINGTON, D. C., SEPT. 5, 6, 7, 8, 9 AND 10, 1887.

### *ON THE CONTRACTIONS OF THE UTERUS THROUGHOUT PREGNANCY, AND THEIR VALUE IN THE DIAGNOSIS OF PREGNANCY, BOTH NORMAL AND COMPLICATED.*

A Paper sent by J. BRAXTON HICKS, M. D., F. R. S., of London, England.

Fifteen years ago, the author had first directed attention to the fact that the uterus contracted throughout pregnancy at intervals of from five to twenty minutes; since then he had added much to his previous knowledge.

Before the fourth month, bimanual palpitation was necessary; later, external examination was sufficient for its detection. The pregnant uterus was very soft, and offered no appreciable resistance to palpitation, except during contraction. In a young girl suspected of pregnancy, abdominal palpitation was often all-sufficient, though internal examination might be necessary. It

*Extracts from advance slips supplied by the *Medical Record*, of New York, from its special report.

was of great advantage to obtain decisive proof before making any allusion to pregnancy. A soft condition of the uterus, with a localized lump, often pointed toward the death of the fœtus or to ectopic gestation. The uterus might contract about fibroids. A knowledge of the contractions often rendered easy a diagnosis otherwise difficult, as in ovarian tumor, ovarian tumor and pregnancy, ectopic gestation and normal gestation, twin pregnancy and hydramnios (palpation and the stethoscope as aids). With a dead fœtus, the walls might be rigidly contracted. We should always look for corroborative signs.

Several cases were then cited in which the diagnosis was rendered certain only by this sign. The conclusions were:

1. That the uterus contracted at intervals of from five to twenty minutes during the whole of pregnancy, remaining contracted for from three to five minutes.

2. The uterus is firm when contracted, and the fœtus cannot be distinctly felt, though, when the uterus is soft, the fœtus is easily mapped out.

3. By noticing the contractions, we are often enabled to diagnose normal pregnancy from other conditions.

4. The contractions have the physiological use of emptying the uterine veins of the carbonized blood.

5. The carbonized blood probably excites the contractions.

Professor Alexander Simpson, of Edinburgh, Scotland, thought the phenomenon of uterine contraction during pregnancy was now a widely-recognized fact. We often meet cases requiring all our diagnostic skill, and should employ all known means. The sign mentioned was especially valuable before the fœtal heart-sounds could be distinguished, and in the third month, when it could be employed in addition to Hegar's sign. One important result of these contractions was that when the uterus contracted forcibly, its contained blood was suddenly emptied into the surrounding parts, distending them, and thus favoring the dilatation of the parturient canal.

Dr. A. F. A. King, of Washington, D. C., said there was sometimes difficulty in recognizing the contractions of the uterus, and they might be excited by polypi, by the retention of menstrual fluid, or by fibroids. They were principally of value after the third month. During the first and second months, we had no positive means of diagnosis. In single women, the diagnosis of pregnancy could not be certainly made by uterine contractions alone. An important point in searching for this sign was to irritate the uterus slightly to make it contract.

Professor Charpentier, of Paris, France, appreciated thoroughly the value of Dr. Hicks' sign, and related a case of hydramnios where its presence made the diagnosis possible.

